# Post-COVID Syndrome: The Research Progress in the Treatment of Pulmonary *sequelae* after COVID-19 Infection

**DOI:** 10.3390/pharmaceutics14061135

**Published:** 2022-05-26

**Authors:** Valentina Ruggiero, Rita P. Aquino, Pasquale Del Gaudio, Pietro Campiglia, Paola Russo

**Affiliations:** 1 Department of Pharmacy, University of Salerno, 84084 Fisciano, Italy; vruggiero@unisa.it (V.R.); aquinorp@unisa.it (R.P.A.); pdelgaudio@unisa.it (P.D.G.); pcampiglia@unisa.it (P.C.); 2 PhD Program in Drug Discovery and Development, University of Salerno, 84084 Fisciano, Italy

**Keywords:** post-COVID syndrome, *long COVID*, inhalation therapy, post-COVID sequelae, post-COVID fibrosis, in vitro lung models

## Abstract

Post-COVID syndrome or *long COVID* is defined as the persistence of symptoms after confirmed SARS-CoV-2 infection, the pathogen responsible for coronavirus disease. The content herein presented reviews the reported long-term consequences and aftereffects of COVID-19 infection and the potential strategies to adopt for their management. Recent studies have shown that severe forms of COVID-19 can progress into acute respiratory distress syndrome (ARDS), a predisposing factor of pulmonary fibrosis that can irreversibly compromise respiratory function. Considering that the most serious complications are observed in the airways, the inhalation delivery of drugs directly to the lungs should be preferred, since it allows to lower the dose and systemic side effects. Although further studies are needed to optimize these techniques, recent studies have also shown the importance of in vitro models to recreate the SARS-CoV-2 infection and study its sequelae. The information reported suggests the necessity to develop new inhalation therapies in order to improve the quality of life of patients who suffer from this condition.

## 1. Introduction

Although most patients have recovered from COVID-19 infections, it has been reported that over 70% of survivors have multiple complications in one or more organs up to 4 months after initial symptoms. The set of long-term consequences caused by the Coronavirus is referred to as post-COVID syndrome or *long COVID* [1].

Even if there are still insufficient data to determine and decisively characterize this syndrome, potential long-term consequences can be assumed from emerging data and previous experiences on other severe respiratory diseases [2].

Survivors of previous coronavirus infections, including the SARS occurrence in 2003 and the Middle East Respiratory Syndrome (MERS) epidemic of 2012, showed a similar set of persistent symptoms, strengthening concerns about the clinically significant sequelae of COVID-19, considering the huge difference in number of patients involved [3,4,5,6].

New research has also demonstrated that the increased risk of sequelae of COVID-19 is independent of age and the presence of previous medical conditions [7] and that patients showed common symptoms such as fatigue, dyspnea, cough, headache, brain fog, anosmia, and dysgeusia. More serious injuries involving the respiratory system have been reported [8].

The lungs are the organs most involved, as the initial site of the infection, with high risk of pneumonia and, in severe cases, acute respiratory distress syndrome (ARDS). In the latter case, patients are often unable to breathe on their own and may require mechanical ventilation to promote the circulation of oxygen in the blood. A well-known sequela of ARDS is pulmonary fibrosis: some of the survivors, in fact, show signs of lung scarring, leading to irreversible impairment of respiratory function [9].

Several active ingredients are capturing the attention of researchers, with the aim to reduce the intensity of symptoms, slowing down the course of the disease, preventing complications and, consequently, improving the quality of life

However, as COVID-19 is a relatively new disease, it is not yet possible to determine which patients are at greater risk for developing long-term lung issues and whether these problems will resolve, improve, or become permanent; it is also not possible to say with high confidence which treatments can prove to be effective against any complications [10]. Given these premises, a series of questions arise:

How much is known about this syndrome? How far are researchers from identifying the active ingredients specifically useful in the treatment of both post-COVID syndrome and aftereffects of infection such as pulmonary fibrosis? Which formulations are considered appropriate, with the lung being one of the most important targets?

The review herein presented aims to face these issues, examining the scientific community reports about the long-term effects of COVID-19 infection and its aftereffects, particularly on the respiratory system and the potential strategies that can be adopted for their management.

## 2. Long-Term Consequences and Aftereffects of COVID Infection

Coronavirus disease 2019 (COVID-19) is caused by a novel coronavirus known as Severe Acute Respiratory Syndrome Coronavirus 2 (SARS-CoV-2) and it was declared a pandemic by the World Health Organization on 11 March 2020 [11].

Coronaviruses are single-stranded, positive-sense RNA viruses that can infect animals and humans. COVID-19 is transmitted between people through small airborne droplets emanated by an infected individual, personal contact (shaking hands), and by touching infected surfaces [12]. This disease often causes no symptoms or mild symptoms in the patients affected by the virus; consequently, they usually have a good prognosis. However, many of these cases develop symptoms in a more severe form that can lead to complications that persist long after the infection [13]. In particular, although COVID-19-associated symptomatology was more evident in individuals with severe disease, individuals with mild and moderate disease also reported a wide range of manifestations after the resolution of the clinical disease [14].

Since the new SARS-CoV-2 is genetically comparable to previously discovered coronavirus strains, such as SARS-CoV and MERS-CoV, it is highly expected that the consequences in patients recovered from COVID-19 are analogous to those of SARS and MERS [15]. Thus, a careful evaluation of the data available in follow-up studies of these infections could provide a useful scenario for identifying effective therapeutic protocols in the treatment of long-COVID syndromes.

SARS-CoV-2 infection mostly affects the respiratory system [8] with complications ranging from mild fatigue to severe forms requiring long-term oxygen therapy or even lung transplantation [16].

The primary pulmonary manifestations of SARS-CoV-2 include hypoxemia, dyspnea, and cough while severe ones include hypoxemic respiratory failure and ARDS. ARDS may progress into pulmonary fibrosis, which in turn leads to irreversible impairment of respiratory function. Respiratory manifestations typical of post-COVID syndrome include chronic cough and persistent dyspnea [17].

Some patients develop important neuropsychiatric and musculoskeletal symptoms of COVID-19 including cerebrovascular accidents, olfactory and gustatory impairments, delirium, and myalgia. Some of the neuropsychiatric and musculoskeletal symptoms of post-COVID syndrome include sleep abnormalities, encephalopathy, chronic headache, delirium, brain fog, and small joint arthritis [18].

**Figure 1:**
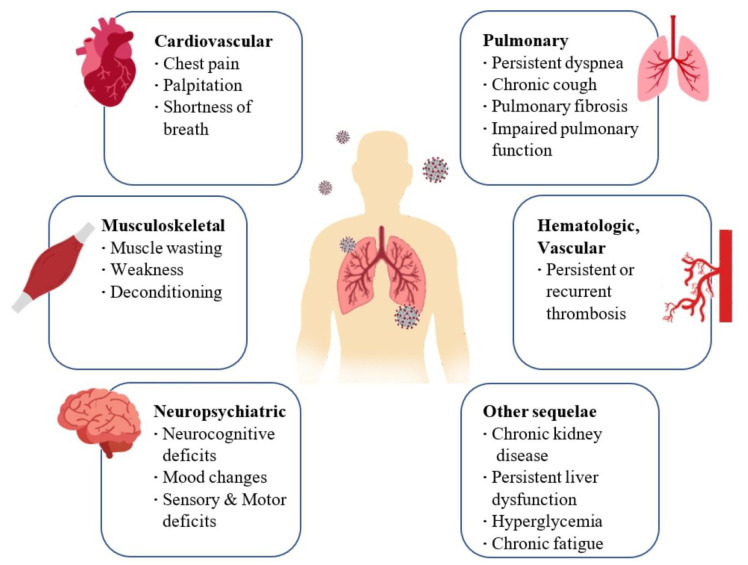
Long-term consequences and aftereffects of COVID-19 infections.

Regarding cardiovascular effects, during the acute phase of the infection, patients may report symptoms of shortness of breath, chest pain, and palpitations. These symptoms may persist up to 6 months after infection. Coagulopathies, thrombotic events that may become recurrent or persistent, hyperglycemia, acute kidney injury, and hepatocellular damage have also been observed (Figure 1) [17,19].

These different COVID-19 symptoms reflect the ability of SARS-CoV-2 to infect different types of human cells [20].

Like other coronaviruses, SARS-CoV-2 shows four structural proteins, known as: S (spike), E (envelope), M (membrane), and N (nucleocapsid) protein. In particular, glycoprotein S is assembled as a homotrimer and is introduced in several copies into the virion membrane, giving it a crown-like appearance. This protein binds the receptor human angiotensin-converting enzyme 2 (ACE2) to infect and enter host cells [20,21].

Although the ACE2 receptor is widely expressed in different organs, its expression level in the airways is of primary interest in the case of COVID-19 pathophysiology.

A recent study on ACE2 expression throughout the respiratory tract revealed that it is greatest in the sinus and alveolar type II cells, allowing for easy entry for SARS-CoV-2 [22].

Moreover, the Ang II/AT1R interaction influences the activation of macrophages that contribute to the so-called cytokine storm [23]. In particular, the ACE2 receptor is a key component of the renin–angiotensin system (RAS). This complex system has a role in the control of blood volume and systemic vascular resistance, which at the same time influences cardiac output and blood pressure [13]. In detail, angiotensinogen is broken down from renin into inactive angiotensin (Ang I), which is converted into angiotensin II (Ang II) by the angiotensin-converting enzyme (ACE). Ang II binds its own AT1R receptor and controls blood pressure and the immune system, stimulating vasoconstriction and inflammation, as well as tissue injury [24].

ACE2 counteracts the activity of ACE by converting Ang I into Ang 1–9 (an inert variety of Ang), but it is also able to break down and hydrolyze the vasoconstrictor Ang II into Ang 1–7, which instead exerts a vasodilator effect [13].

**Figure 2:**
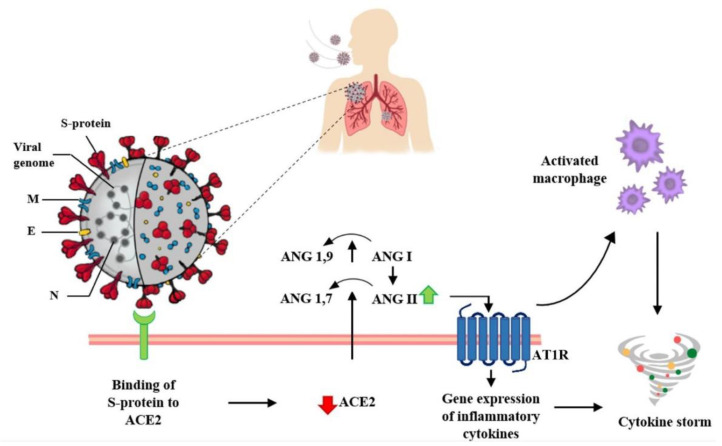
Interaction between SARS-CoV-2 and the Renin–Angiotensin System. SARS-CoV-2 enters host cells through the interaction of its spike protein with the ACE2 receptor. The downregulation of ACE2 receptors results in a decrease in the cleavage of angiotensin I and angiotensin II at Ang 1–9 and Ang 1–7, respectively. Ang II, through interaction with the AT1R receptor, stimulates the gene expression of various inflammatory cytokines and also influences the activation of macrophages that contribute to the “cytokine storm”.

Therefore, the downregulation of ACE2 receptors due to binding with the viral spike protein leads to an increase in angiotensin II, with consequent harmful pro-inflammatory effects. Ang II, in fact, by interacting with its AT1R receptor, stimulates the gene expression of various inflammatory cytokines [23] (Figure 2).

This cytokine storm has been hypothesized to contribute to the development of acute respiratory distress syndrome (ARDS) after COVID-19 infection [25]. In fact, it has been observed that patients with severe manifestations of COVID-19 often progress to ARDS with permanent scarring of lung tissue and respiratory issue persisting extensively after recovery [2,26]. In several autopsy reports, bilateral diffusion of alveolar damage with fibromyxoid cell exudates, pneumocytes desquamation, and hyaline membrane formations have been observed [27].

The pathological evolution of ARDS consists of three phases: exudative, proliferative, and fibrotic. In the exudative phase, the extra presence of proinflammatory cytokines (IL-1β, TNF, and IL-6) leads to the influx of neutrophils into the lung tissue and the breakdown of the endothelial–epithelial barrier, with consequent loss of fluids in the alveolar spaces and respiratory distress. This phase is followed by the fibroproliferative phase, in which fibrocytes, fibroblasts, and myofibroblasts accumulate in the alveolar compartment, leading to excessive deposition of extracellular components of the matrix (ECM) including fibronectin, collagen I, and collagen III [28], in order to promote tissue repair.

Although mechanical ventilation (MV) is the most important adjuvant therapy for ARDS, it can worsen lung damage [29] since besides inducing the secretion of transforming growth factor β1, it also activates collagen synthesis and inhibits collagenase production [30]. A further problem following mechanical ventilation is respiratory muscle dysfunction: respiratory muscle weakness is approximately two times limb muscle weakness after 1 day of mechanical ventilation, and sepsis, muscle immobilization, and steroids contribute to weakness acquired in intensive care units (ICU) [31].

A fraction of survivors from ARDS progress to pulmonary fibrosis, which is characterized by the inability of the lungs to rebuild the damaged alveolar epithelium, persistence of fibroblasts, and disproportionate deposition of collagen and other extracellular components of the matrix [32]. Normally, once the normal lung architecture is rebuilt, the temporary ECM is removed and the fluid from pulmonary edema in the alveolar areas is eliminated as well. However, if ARDS is not managed quickly enough, persistent lung damage will drive uncontrolled fibroproliferation through upregulation of the profibrotic pathways and downregulation of the antifibrotic pathways: among the various profibrotic pathways, transforming growth factor-beta (TGF-β) is the most important mediator and its expression is effectively upregulated in the lungs following SARS-CoV-2 infection [22,33].

**Figure 3:**
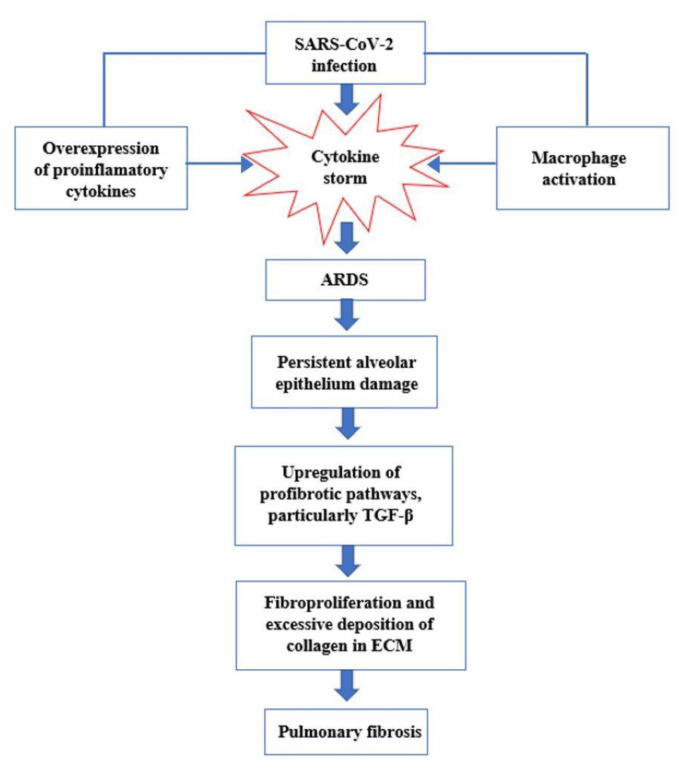
Key events in the progression of cytokine storm to acute respiratory distress syndrome (ARDS) and pulmonary fibrosis.

The activation of TGF-β leads to the deposition of extracellular matrix proteins, stimulation of fibroblast chemotactic migration, and fibroblast to myofibroblast transition (Figure 3) [10].

It is also suggested that following the dysregulation in immunological mechanisms developed as a consequence of COVID-19, an immunosuppressive state occurs to avoid progression to organ damage, especially after the acute hyperinflammatory phase. A prolonged stage of immunosuppression, however, can increase the risk of secondary bacterial and fungal infections [28,34].

A significant proportion of survivors from COVID-19 infection showed impaired lung function 6 months after recovery. This is important, not only for the long-term follow-up of these patients, but also to underline the persistent respiratory failure that can result from SARS-CoV-2 infection. Studies of previous coronavirus infections indicate that patients may develop a permanent impairment that lasts for months or even years after infection [6,35,36]. Among the results of the pulmonary function tests, the decrease in the diffusion capacity of carbon monoxide was more evident [37]. Weakness of the respiratory muscles, development of fibrosis, thrombosis, and angiopathies, particularly those associated with previous diseases and follow-up processes in intensive care units, are just some of the risk factors leading to a worsening of lung function [34,36].

## 3. Management of Patients with Post-COVID Syndrome and after Effects of SARS-CoV-2 Infection

Considering the events occurring after the infection, several classes of active ingredients may be useful in relieving the effects of infection in the airways (Table 1):

Mast cell level stabilizers: mast cell level stabilizers, can inhibit an important increase in post-COVID-19 disease, which, in a considerable fraction of patients, can be driven by persistent chronic mast cell activation [38]. Several studies have shown a potential association of mast cells with COVID-19 [38,39,40,41]. It is known that the activation of mast cells located in the submucosa of the respiratory tract leads to the actualization of pro-inflammatory cytokines such as IL-1, IL-6, and TNF-α, contributing to the development of the cytokine storm. Furthermore, autopsy results of the lungs of patients who died from COVID-19 showed an accumulation of mast cells, which has been hypothesized to be the cause of pulmonary edema, inflammation, and thrombosis in the pathophysiology of COVID-19.

Examples of mast cell stabilizers are flavonoids: these are a large group of natural stabilizers, including luteolin, apigenin, kaempferol, fisetin, quercetin, genistein, and epigallocatechin gallate. Some antihistamine drugs have also shown anti-inflammatory and mast cell-stabilizing effects such as olopatadine, rupatadine, and ketotifen [40,42,43,44].

Even clarithromycin, the antibacterial belonging to the macrolide group, has been shown to be a highly effective mast cell stabilizer [41,45]: its dual ability as a mast cell stabilizer and an antibiotic, useful in cases of bacterial superinfections in the lungs, makes it a promising candidate for the treatment of long COVID patients. For this reason, its formulation as an inhalation product [46,47] can be of considerable interest to increase the weapons available to fight the typical effects of COVID infections.

Anti-inflammatory drugs: Most patients who develop post-COVID 19 interstitial lung disease (PC ILD) are hypoxic, and steroids have become the standard of care in hypoxic patients in intensive care units around the world after the results of the recovery trial [48]. Generally, the oral administration of 4–6 mg of dexamethasone for no more than 10 days is suggested [10] since the use of high doses of oral steroids is associated with adverse effects such as hyperglycemia, delayed viral clearance, and increased susceptibility to infections as well as thromboembolic diseases [49].

However, the role of inhaled corticosteroids in the treatment of patients with mild to moderate COVID-19 disease is less clear. Inhaled corticosteroids are drugs that are breathed into the lower airways through an inhaler where they reduce inflammation in the lungs. They are generally used to heal respiratory diseases like asthma and chronic obstructive pulmonary disease. Long-term use and improper inhaler method may lead to side effects that include a mouth infection called thrush, a change in voice, and an increased risk of lung infections [50]. Inhaled corticosteroids reduce the expression of ACE2 [51]. It has been shown that ciclesonide inhibits the enzyme PAK1, a pathogenic pathway for COVID-19 related to ACE2: by inhibiting PAK1, ciclesonide reduces immune suppression and reduces lung inflammation [52]. A phase 3, multicenter, double-blind, randomized clinical trial was aimed at 10 centers in the USA and estimated the safety and efficacy of a ciclesonide metered-dose inhaler (MDI) for treating hospitalized participants with symptomatic COVID-19 infection (from 11 June 2020 to 3 November 2020). A total of 400 participants were enrolled and randomized: 197 patients receive ciclesonide MDI, 160 μg per actuation, for a total of 2 actuations twice a day (total daily dose, 640 μg) and 203 receive placebo for 30 days. In this randomized clinical trial, ciclesonide reduced the time to alleviate all COVID-19-associated symptoms. Patients who were cured with ciclesonide had fewer consequent emergency department visits or hospital admissions for reasons that were connected to COVID-19. These results suggest that future research on inhaled steroids is needed to explore their efficacy in patients with a high risk for disease progression and in reducing the incidence of long-term COVID-19 symptoms [53].

Antibiotics: SARS-CoV-2 can directly damage the lung epithelium and indirectly trigger the cytokine storm, ultimately leading to multi-organ failure. To counteract the excessive and dysregulated response of the immune system, immunosuppressive drugs are widely used. The combination of these drugs with virus-induced immunosuppression can increase predisposition to secondary infections [54]. Many patients with severe COVID-19 have received empiric antibiotics to prevent secondary bacterial infections. However, this solution raises concerns about antibiotic abuse and the consequent harm associated with bacterial resistance that could be considered a further long-term complication of the SARS-CoV-2 pandemic [55].

It has been reported that antibiotics, in addition to preventing and treating bacterial infections, may also have interesting antiviral and immunomodulatory properties in the treatment of COVID-19. This is the case of azithromycin, an antibiotic belonging to the class of macrolides, which could play a fundamental role in the hyperinflammatory phase of COVID-19 thanks to its ability to downregulate the production of cytokines, maintain epithelial integrity, and prevent pulmonary fibrosis, which is one of the most serious after-effects of infection [56,57]. In fact, one of the mechanisms of action proposed for the treatment of COVID-19 concerns precisely the ability of azithromycin to inhibit the proliferation of fibroblasts, reduce the production of collagen and the levels of TGF-β (the most important path in the development of fibrosis) and, finally, inhibit the stimulation of the pro-fibrotic gene induced by TGF-β. However, although azithromycin represents a promising therapy, data on its use in COVID-19 are still insufficient [57].

Antifibrotic agents: Emerging data from the COVID-19 pandemic suggest that there may be major fibrotic consequences following SARS-CoV-2 infection. Since transforming growth factor-β (TGF-β) is the most important pathway in the development of fibrosis and is upregulated in SARS-CoV-2 infection, antifibrotic agents could prove to be a valid treatment.

In particular, pirfenidone is an oral antifibrotic agent [58] capable of blocking the synthesis of collagen induced by TGF-β, by inhibiting the activation of the HSP47 and Col1 genes. The anti-inflammatory effect is given by suppressing the production of tumor necrosis factor-α (TNF-α), interferon gamma (IFN-γ), interleukin-1beta (IL-1β), and interleukin-6 (IL-6). In addition, it has antioxidant properties: depending on the concentration, it blocks NADPH-dependent microsomal lipid peroxidation in the liver [59]. On the basis of in vivo models, it has been determined that pirfenidone suppresses the differentiation of TGF-β-associated fibroblasts in the lungs [60]. A recent study on the use of pirfenidone in patients with severe Coronavirus Disease 2019 (NCT04282902) was conducted from 31 January to 3 March 2020 at Tongji Hospital and Jingzhou Hospital, and was approved by the Institutional Review Committee of Tongji Hospital and Jingzhou Hospital. A total of 146 patients were randomly assigned in a 1:1 ratio to pirfenidone (200 mg, three times daily for the first two days and 400 mg, three times daily thereafter) plus standard therapy or standard therapy alone. Pirfenidone was given through a nasogastric tube to patients who were unable to swallow.

This study showed that pirfenidone reduced the cytokine storm, responsible for complications in patients with severe COVID-19 [61].

Curcumin, a naturally occurring polyphenolic compound with antifibrotic properties, has been demonstrated to be effective in significantly decreasing the expression of the TGF-β II receptor (TGF-ß RII), as well as in directly reducing the expression of the TGF-β protein and its mRNA [62].

Since the therapeutic application of curcumin is still limited by its poor oral bioavailability, hydrophobicity, and rapid metabolism in intestine and liver, researchers are studying new formulations including curcumin nanoformulations, which refer to the process of complexing curcumin with small nanoscale-sized molecules.

Nanoparticle formulation as a drug delivery method has proven to be revolutionary because it can stabilize an active compound in a physiological environment, increasing its cellular uptake and bioavailability, and eventually making the drug more effective.

In addition, curcumin nanoformulations can be generated as formulations for inhalation and rapidly administered to patients with COVID-19 ARDS undergoing mechanical ventilation. The optimal dose of curcumin must be high enough to exert the therapeutic effect, even if a dose greater than 7500 mg/day can actually facilitate the entry of SARS-CoV-2 by upregulating ACE2 expression. Although they have shown promising potential, further studies on the recommended dosage of curcumin aerosols administration, the choice of the inhalation device, and the long-term effects on the lungs [22] are still needed.

Antioxidants: since respiratory viruses not only stimulate ROS production but also damage cellular defense systems, the application of radical scavengers is proposed as a possible therapeutic approach. In this sense, the most encouraging compounds include GSH, its precursor N-acetylcysteine (NAC), and natural molecules such as flavonoids [13].

A recent phase II study (NCT04374461) still in progress aims at evaluating the effect of NAC (iv; 6 g/day) administration in patients with severe symptoms of COVID-19; its results will help to clarify the potential therapeutic properties of this drug in patients with COVID-19 [63].

Moreover, a case report study showed that both oral and intravenous administration of 2000 mg GSH was effective in mitigating severe respiratory symptoms of COVID-19, demonstrating the usefulness of this antioxidant therapy for patients with COVID-19 [64].

Antivirals: there is a close relationship between the appearance of pulmonary fibrosis and high viral load [34].

Recently, in the UK the oral antiviral agent molnupiravir has been approved, a potent ribonucleoside analogue that inhibits the replication of SARS-CoV-2, acting on the enzyme that the virus uses to generate copies of itself by introducing errors into its genetic code. Furthermore, the data indicate that molnupiravir reduced the risk of hospitalization or death in approximately 50% of adults who were at risk of a severe disease outcome [65]. The molnupiravir was authorized for distribution in emergency conditions by a Decree of the Ministry of Health on 26 November 2021 and is indicated within 5 days of the onset of symptoms. The duration of treatment is 5 days and consists of taking four tablets (from 200 mg) two times a day. [66].

New therapeutic agents: considering the extent and impact of the pandemic, there is an immediate necessity to evaluate also new experimental drugs with a biological rationale for use in post-COVID syndrome with effects on the airways [10].

In this regard, a study on the experimental use of Zofin in a patient who exhibited the classic symptoms of post-COVID-19 syndrome including headache, chest tightness, brain fog, fatigue, fever, and shortness of breath was presented. The patient was treated at Advanced Regenerative Therapies in Savanah, GA. Three 1 mL doses of Zofin were administered intravenously on Day 0, 4, and 8 in an outpatient setting. The treating physician followed up with the patient daily during treatment and continued to monitor progress on follow-up visits; Day 14, 21, 28, and 60. The patient was monitored for 30 min immediately following the IV infusion. At the end of the study, the intravenous and multidose administration of the drug was revealed to be safe and well-tolerated without any serious adverse events. Moreover, the patient reported no signs of respiratory distress or other disorders. On 14 April 2022, a phase I/II randomized double-blinded and placebo control trial was started, with the aim to assess the safety and potential efficacy of Zofin administered intravenously in 30 subjects experiencing prologue symptoms. Results from September 2023 (NCT05228899). Zofin is an acellular biological therapy that derives from the soluble and nanoparticle fraction of human amniotic fluid and is produced to retain naturally occurring microRNAs, growth factors, cytokines, and chemokines, as well as extracellular vesicles and exosomes secreted by perinatal tissues [67]. Therefore, this therapy proposes the use of paracrine factors derived from cells and secreted by tissues, rather than the cells themselves, as active components of the drug. In fact, although cell therapies are actively tested for COVID-19 infections due to the observed immunomodulatory and anti-inflammatory effects, they have several limitations such as inadequate cell survival after infusion [68]. Extracellular vesicles, carriers of proteins and nucleotides, have shown anti-inflammatory and tissue regenerative effects in various preclinical models and have been shown to be useful in alleviating symptoms associated with respiratory distress and in promoting endogenous tissue repair through alveolar tissue repair and the modulation of inflammatory immune cells. The promising results reported in this study encourage further studies [67].

Once the active ingredient to be administered, or a mixture of active ingredients in the case of polytherapy, has been identified, it is of fundamental importance to develop a formulation capable of delivering the active compound directly to the site of action, at the level of the airways, with a reduction in dosage and, consequently, of side effects.

**Table 1:** Different active ingredients useful in the treatment of post-COVID sequelae.

Drug	Category	Mode of Action	References
Flavonoids (luteolin, apigenin, kaempferol, fisetin, quercetin, genistein, and epigallocatechin gallate)	Mast cell level Stabilizers	Anti-inflammatory and mast cell-stabilizing effects	[40,42,43]
Antihistamine drugs (olopatadine, rupatadine, and ketotifen)	Mast cell level Stabilizers	Anti-inflammatory and mast cell-stabilizing effects	[40,42,44]
Clarithromycin	Mast cell level Stabilizers	Anti-inflammatory and mast cell-stabilizing effects	[41,45]
Dexamethasone	Corticosteroids	Decreases the inflammation linked with cytokine release syndrome	[48]
Ciclesonide	Corticosteroids	Anti-inflammatory action	[53]
Azithromycin	Antibiotics	Inhibit the proliferation of fibroblasts, reduce the production of collagen and the levels of TGF-β	[57]
Pirfenidone	Antifibrotic	Inhibit the synthesis of collagen induced by TGF-β; suppresses the production of TNF-α, IFN-γ, IL-1β- and IL-6; suppresses the differentiation of fibroblasts associated with TGF-β	[61]
Curcumin	Antifibrotic	Decreasing the expression of the TGF-β II receptor (TGF-ß RII), as well as in directly reducing the expression of the TGF-β protein and its mRNA	[22]
N-Acetylcysteine (NAC)	Antioxidants	Inhibits virus replication and expression of pro-inflammatory molecules. Boosting a type of cell in the immune system that attacks infections	[63]
GSH	Antioxidants	Blocks viral replication through redox state modulation	[64]
Molnupiravir	Antivirals	Inhibits the replication of SARS-CoV-2, acting on the enzyme that the virus uses to generate copies of itself by introducing errors into its genetic code	[66]
Zofin	Derived from human amniotic fluid	Suppressor of cytokine activation	[67]
Ampion	Biological Drug	Modulate inflammatory cytokine levels	[69]

## 4. Post-COVID Inhalation Therapy

The SARS-CoV-2 virus is transmitted through the respiratory system and, as mentioned above, can cause an excessive and dysregulated response of the immune system with consequent damage to the lungs [12,70]. Since the main site of infection and disease progression is the upper and lower airways [33,71], and a close relationship between the development of pulmonary fibrosis and high viral load has been shown [72], inhaled administration of an anti-COVID-19 drug is favored as it allows for a high concentration of the active ingredient where the viral load is highest. In fact, in viral diseases that mainly affect the lungs, oral drugs, and parenteral applications have shown several limits including low drug concentration at the desired site, while increasing the drug dose to reach the necessary concentration leads to intolerable side effects. Conversely, inhaled administration of the drug would not only directly affect the lungs, but would also require a lower dose, reducing the risk of systemic side effects [73]. This should also make the treatment more effective and tolerable.

Additional advantages over other routes are direct administration to the target site and rapid onset of action [12,74], as well as self-administration [75], improved patient compliance [76], and non-invasive nature [77].

In the case of a lung formulation, bioavailability requires that the dose of medication be deposited in the lower respiratory tract [78]. Failure to give evidence can lead to ineffectiveness. The transport in the upper airways is limited by a smaller surface area and lower regional blood flow. In contrast, the smaller airways and alveolar space account for more than 95% of the lung’s total surface area, and this compartment is directly connected to the systemic circulation via the pulmonary circulation [79].

However, inhalation may be complex for a number of reasons:

Firstly, the lung should not be considered as a uniform organ, its fine and branched architecture hinders the deep deposition of the drug [80].

Though, the pharmacological response depends not only on the number of particles deposited, but also on the amount retained at the site [81]. In fact, the respiratory tract has developed defense mechanisms with the aim of keeping inhaled materials out of the lungs, as well as removing or inactivating them once deposited [82].

Secondly, it is necessary for the patient to use a suitable inhaler device, which is comfortable and discreet but above all easy to use, as it is precisely on the correct use of the devices that partly depends on the success of the therapy [83]. According to the European Pharmacopoeia, there are three devices that can be used to release a drug into the lungs:

Nebulizers: Nebulizers operate by atomizing the bulk liquid formulation into fine droplets for inhalation. Most nebulizers use compressed air for atomization or ultrasonic energy [84]. They are usually cumbersome and inconvenient to carry and operate. Some well-documented disadvantages include high cost, low efficiency, poor reproducibility and high variability, risk of bacterial contamination, and constant cleaning requirements [85]. Moreover, aerosol administration via nebulization is time consuming; approximately 30 min if set-up, drug administration, and cleaning are taken into account [86].

Pressurized metered-dose inhalers (pMDI): A pMDI is a pressurized metal canister containing a mixture of propellants, surfactants, preservatives, and drug. The drug represents about 1% of the contents, while the propellants are greater than 80% of the contents, by weight [87,88]. The pMDI is popular because it is compact, portable, cost-effective, and provides repeated dosing (100 to 400 actuations) [89]. The effective use of pMDIs, however, requires an adequate coordination between inspiration and actuation of the inhalers, and many patients and healthcare providers are not able to demonstrate the correct pMDI technique [90,91,92,93,94,95].

Dry powder Inhalers (DPI): DPIs offer the opportunity to use solid-state formulations for aerosol delivery to the lungs [96,97].

These devices are very portable, easy to use, and do not require spacers. Unlike pMDI, they are ‘breath-actuated’ devices delivering the drug only when required by patient inhalation eliminating the problem of coordination between device activation and patient inspiration [98,99]. In addition, DPIs do not require propellant gases, reducing the costs associated with the generation, transport, and storage of propellant and their unwanted environmental impact [100]. However, there is a minimum amount of energy, hence inhalation flow, necessary to obtain efficient disaggregation of the formulation: very young and elderly patients and those experiencing a severe exacerbation may not be able to generate inhalation flow sufficient to produce turbulent energy that produces a dose reaching the lungs from some devices [101].

Recently, Ampio Pharmaceuticals launched a phase I randomized study to evaluate the safety, tolerability, and efficacy of nebulized Ampion in improving the clinical outcomes of 40 patients hospitalized with COVID-19 infections, with persistent respiratory symptoms.

Details on the study will be published as soon as they are ready [69] Ampion is the low molecular weight filtrate of human serum albumin and as an immunomodulatory agent with anti-inflammatory effects; it has the potential to modulate inflammatory cytokine levels related to COVID-19 disease and respiratory complications, such as respiratory distress syndrome. acute (ARDS). Administration of Ampion to patients by inhalation allows the drug to reach the target site directly and attenuate lung inflammation [12].

## 5. In Vitro Models for the Study of Post-COVID Syndrome Drugs

The health *sequelae* that patients encounter once recovered from COVID-19 have pushed scientists and pharmaceutical industries all over the world to develop new drug products able of countering the issues produced by the current pandemic.

To this aim, while a number of preclinical cell culture techniques have been used to solve issues related to the pathogenesis of SARS-CoV-2, viral replication mechanisms, the use of in vitro model to study the efficacy of active ingredients in post-COVID sequelae is still far behind [102].

One of the main problems in the development of drugs against coronavirus and its sequelae in cell cultures, in fact, is the poor predictability of their effectiveness within the human body.

Traditional monolayer culture systems fail to mimic the behavior of the respiratory system in vivo. In these models, the cells are grown on a hard, flat, two-dimensional (2D) surface, so it is not possible to model lung fibrosis [103], the most serious consequence after the infection. The results obtained using animal models, on the other hand, are not reliable due to the fundamental differences in lung architecture and in the immune response observed in different species [103,104].

Unfortunately, it can take years for studies of large patient cohorts to provide sufficient information on the clinical course of fibrotic remodeling. Therefore, it is essential to study post-COVID pulmonary fibrosis in models in which SARS-CoV-2 infection can be reproduced in order to recreate an environment as similar as possible to the real one.

Some of the in vitro models that can be successfully employed for this purpose include precision-cut lung slices (PCLS), pulmonary organoids, and lung-on-chip (LOC) devices.

Precision-cut lung slices (PCLS) are gaining attention as a new ex vivo model for pulmonary fibrosis. These systems, in fact, are able to mimic changes due to fibrosis, including greater deposition of extracellular matrix and alveolar remodeling when induced with profibrotic factors [105].

A key advantage of PCLS is the maintenance of a lung 3D structure that allows the analysis of the spatial and functional relationships of cells in the entire alveoli and airways. Furthermore, as multicellular systems, they preserve most of the functional cells of the lungs allowing for an accurate representation of the native biological environment of the cells, overcoming the limits in the cell–cell and cell–matrix interaction of most approaches of 2D cell culture [106].

The PCLTS protocol requires fresh tissue, unlike decellularized matrices that can be frozen and stored for later use. Human lung tissue (from cadavers or surgical resections) is filled with low melting point agarose and then cut into 300–1000 μm thick slices for in vitro culture. Lung slices prepared using this protocol were shown to be viable for 5 days, while the ciliary beating of bronchial epithelial cells was documented on day 7, confirming the preservation of cell function in these matrices for up to 1 week [107].

Even if it is possible to obtain slices of uniform thickness, the type and number of specific cells can vary from slice to slice, especially if there are irregularly distributed disease-related changes. In general, PCLS can be considered a “mini” lung [108], useful for studying specific aspects of pulmonary fibrosis and viral infection directly in human lung tissue.

Although human proximal and distal airway lung explants have been shown to be predisposed to SARS-CoV-2 infection [109], this technique has not yet been used to investigate COVID-19 and its sequelae [105]. Several disadvantages, in fact, counteract its use: PCLS mainly allow the study of local immune responses, while the infection processes involve both local and systemic immune responses. Culture in an air–liquid interface and the addition of specific immune cells could improve this system [110]. The short culture period and the limited and unpredictable availability of fresh human lung tissue further prevent its use [111].

Over the past year, organoids have also begun as potent tools for COVID-19 research. Organoids are three-dimensional tissue cultures resulting from pluripotent or induced pluripotent stem cells (PSC/iPSC) that self-organize spatially in a similar way to their in vivo counterparts. Stem cells are seeded on collagen suspended clusters or ECM solution such as Matrigel (an ECM isolated from Engelbreth-Holm-Swarm (EHS) mouse sarcoma cells) with the addition of appropriate growth factors for differentiation into the lineage of interest [112].

These models have the potential to overcome the limitations of conventional cell cultures or animal models due to their high similarity to the human organ, thus providing a reliable in vitro platform to study the mechanisms and pathogenesis of viral diseases [102].

In fact, they authentically reproduce cell–cell and cell–matrix interactions, can be kept in culture for several weeks, and have been shown in various studies to provide an acceptable model of SARS-COV-2 infection in the alveoli, which is also useful for studying the sequelae resulting from this infection [105].

This technique has already been used in the past to study pulmonary fibrosis: pulmonary organoids derived from human pluripotent stem cells (hPSC) containing a mix of epithelial and mesenchymal cells have been genetically modified to develop Hermansky–Pudlak syndrome, a condition clinical comparable with IPF, with the aim to identify new potential drug targets [105,113].

Despite the numerous advantages of these models, organoids are not ideal for generating complex co-cultures mimicking the different structures and functions of organs and tissues.

Further limitations are due to the lack of vascularization and air–liquid interface, without taking into account the high variability of organoids in terms of size and cellular composition [112].

A higher degree of complexity can be achieved in long-on-chip (LOC) models, which are part of a broader category of models called organ-on-chip that simulate the activities, mechanics, and physiological response of entire organs through the modulation of a variety of biological, physical, and chemical factors in a controlled microenvironment [114]. Having a controlled and dynamic system is essential to quickly test drugs safely, especially when dealing with dangerous pathogens such as the coronavirus.

Lung-on-chips, in particular, aim to closely mimic the functionality of the lungs and gas exchanges in the alveoli.

In 2010, an organ-on-chip model of the lung alveolus was generated, in which two adjacent channels were separated by a porous polydimethylsiloxane (PDMS) membrane. After coating each side of the membrane with an extracellular matrix, solutions containing lung epithelial cells or vascular endothelial cells were introduced into the channels, allowing the cells to expand on both sides of the membrane. After confluence, the growth medium from the upper canal was removed to generate an air–liquid interface.

**Figure 4:**
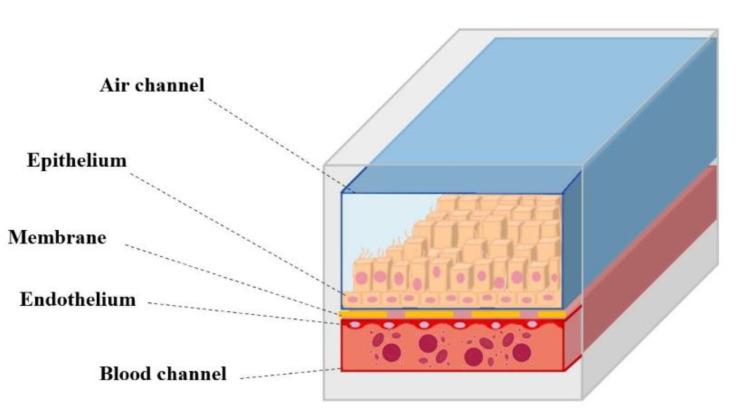
Schematic representation of the lung-on-chip device: in the microchannel in red flows a liquid similar to blood, in the blue one the air flows.

Therefore, this microfluidic technology allows the reproduction of cellular interactions in a vascularized environment, closely mimicking the function of the organ (Figure 4) [112].

In fact, the integration with a biological-inspired mechanical driven system that uses computer-controlled negative pressure to cyclically lengthen the alveolar–capillary barrier, allows for simulating physiological respiratory movements. Importantly, this device displays responses not normally recorded in traditional cell culture models, such as the recruitment and phagocytic activity of immune cells in response to bacteria, inflammatory cytokines, and environmental nanoparticles. Furthermore, the ability of this model to reproduce lung function led to the discovery of negative effects induced by the respiratory act, previously considered inexplicable [105,115].

In the past, this device has been used to study influenza virus infections [116], thus paving the way for further in vitro studies, including research on SARS-CoV-2 and its sequelae. In fact, several studies have reported that these models are suitable for studying SARS-CoV-2 infection since they are able to reproduce the function and lesion of the capillary–alveolar barrier, SARS-CoV-2 infection, the resulting inflammatory response, and the recruitment of immune cells [102,117,118]. Furthermore, compared to conventional cell culture methods, LOCs are stable for a longer period of time, and this is particularly important for the study of diseases such as pulmonary fibrosis [107].

However, the fabrication of long-on-a-chip models is quite expensive, limiting the applications of this technique [119].

## 6. Conclusions

Post-COVID syndrome is a new condition that can adversely affect quality of life, regardless of age and the presence of pre-existing diseases. Unfortunately, it is not yet possible to know which patients are most at risk of developing long-term consequences and whether these problems will solve, improve, or become permanent. This review examined the reports of the scientific community on the long-term consequences of COVID-19 and its after-effects, particularly in the lung, as the main site of infection, and possible treatment options useful for alleviating its symptoms. Active ingredients demonstrating a biological logic in the treatment of post-COVID sequelae have been reported, concluding that the most appropriate type of formulation for their administration is inhalation, allowing for the release of the drug directly on the site of action with a reduction in dose and systemic side effects. Considering also that pulmonary fibrosis has been reported as one of the most serious consequences, the development of new in vitro experimental models, able to faithfully recreate the infection, will help scientists and pharmaceutical companies around the world to develop therapeutic strategies for similar conditions; although, further studies are needed to overcome the limitations of these techniques.

## Data Availability

Not applicable.

## References

[1] Yan Z., Yang M., Lai C.-L. (2021). Long COVID-19 syndrome: A comprehensive review of its effect on various organ systems and recommendation on rehabilitation plans. Biomedicines.

[2] Jiang D.H., McCoy R.G. (2020). Planning for the post-COVID syndrome: How payers can mitigate long-term complications of the pandemic. J. Gen. Intern. Med..

[3] Nalbandian A., Sehgal K., Gupta A., Madhavan M.V., McGroder C., Stevens J.S., Cook J.R., Nordvig A.S., Shalev D., Sehrawat T.S. (2021). Post-acute COVID-19 syndrome. Nat. Med..

[4] Hackett M.L., Glozier N.S., Martiniuk A.L., Jan S., Anderson C.S. (2011). Sydney Epilepsy Incidence Study to Measure Illness Consequences: The SESIMIC Observational Epilepsy Study Protocol. BMC Neurol..

[5] Ahmed H., Patel K., Greenwood D., Halpin S., Lewthwaite P., Salawu A., Eyre L., Breen A., O’Connor R., Jones A. (2020). Long-Term Clinical Outcomes in Survivors of Severe Acute Respiratory Syndrome and Middle East Respiratory Syndrome Coronavirus Outbreaks after Hospitalisation or ICU Admission: A Systematic Review and Meta-Analysis. J. Rehabil. Med..

[6] Hui D.S. (2005). Impact of Severe Acute Respiratory Syndrome (SARS) on Pulmonary Function, Functional Capacity and Quality of Life in a Cohort of Survivors. Thorax.

[7] Daugherty S.E., Guo Y., Heath K., Dasmariñas M.C., Jubilo K.G., Samranvedhya J., Lipsitch M., Cohen K. (2021). Risk of clinical sequelae after the acute phase of SARS-CoV-2 infection: Retrospective cohort study. BMJ.

[8] Garg M., Maralakunte M., Garg S., Dhooria S., Sehgal I., Bhalla A.S., Vijayvergiya R., Grover S., Bhatia V., Jagia P. (2021). The conundrum of ‘Long-COVID-19’: A narrative review. IJGM.

[9] Michalski J.E., Kurche J.S., Schwartz D.A. (2022). From ARDS to pulmonary fibrosis: The next phase of the COVID-19 pandemic?. Transl. Res..

[10] Udwadia Z., Koul P., Richeldi L. (2021). Post-COVID lung fibrosis: The tsunami that will follow the earthquake. Lung India.

[11] Boechat J.L., Chora I., Morais A., Delgado L. (2021). The immune response to SARS-CoV-2 and COVID-19 immunopathology—Current perspectives. Pulmonology.

[12] Eedara B.B., Alabsi W., Encinas-Basurto D., Polt R., Ledford J.G., Mansour H.M. (2021). Inhalation delivery for the treatment and prevention of COVID-19 infection. Pharmaceutics.

[13] Fratta Pasini A.M., Stranieri C., Cominacini L., Mozzini C. (2021). Potential role of antioxidant and anti-inflammatory therapies to prevent severe SARS-CoV-2 complications. Antioxidants.

[14] Salamanna F., Veronesi F., Martini L., Landini M.P., Fini M. (2021). Post-COVID-19 Syndrome: The Persistent Symptoms at the Post-Viral Stage of the Disease. A Systematic Review of the Current Data. Front. Med..

[15] Sivashanmugam K., Kandasamy M., Subbiah R., Ravikumar V. (2021). Repurposing of histone deacetylase inhibitors: A promising strategy to combat pulmonary fibrosis promoted by TGF-β signalling in COVID-19 survivors. Life Sci..

[16] Ali R.M.M., Ghonimy M.B.I. (2021). Post-COVID-19 pneumonia lung fibrosis: A worrisome sequelae in surviving patients. Egypt. J. Radiol. Nucl. Med..

[17] Lutchmansingh D.D., Knauert M.P., Antin-Ozerkis D.E., Chupp G., Cohn L., Dela Cruz C.S., Ferrante L.E., Herzog E.L., Koff J., Rochester C.L. (2021). A clinic blueprint for post-coronavirus disease 2019 RECOVERY. Chest.

[18] Mehandru S., Merad M. (2022). Pathological Sequelae of Long-Haul COVID. Nat. Immunol..

[19] Rezkalla S.H., Kloner R.A. (2021). Post-acute sequelae of SARS-COVID-2 syndrome: Just the beginning. Cardiol Res.

[20] Proal A.D., VanElzakker M.B. (2021). Long COVID or post-acute sequelae of COVID-19 (PASC): An overview of biological factors that may contribute to persistent symptoms. Front. Microbiol..

[21] Jackson C.B., Farzan M., Chen B., Choe H. (2022). Mechanisms of SARS-CoV-2 entry into cells. Nat. Rev. Mol. Cell Biol..

[22] Yo E.C., Kadharusman M.M., Karman A.P., Louisa M., Arozal W. (2021). Potential pharmacological options and new avenues using inhaled curcumin nanoformulations for treatment of post-COVID-19 fibrosis. Syst. Rev. Pharm..

[23] Banu N., Panikar S.S., Leal L.R., Leal A.R. (2020). Protective role of ACE2 and its downregulation in SARS-CoV-2 infection leading to macrophage activation syndrome: Therapeutic implications. Life Sci..

[24] Ramasamy S., Subbian S. (2021). Critical determinants of cytokine storm and type I interferon response in COVID-19 pathogenesis. Clin. Microbiol. Rev..

[25] Channappanavar R., Perlman S. (2017). Pathogenic human coronavirus infections: Causes and consequences of cytokine storm and immunopathology. Semin. Immunopathol..

[26] Thompson B.T., Chambers R.C., Liu K.D. (2017). Acute Respiratory Distress Syndrome. N. Engl. J. Med..

[27] Suess C., Hausmann R. (2020). Gross and histopathological pulmonary findings in a COVID-19 associated death during self-isolation. Int. J. Leg. Med..

[28] Oronsky B., Larson C., Hammond T.C., Oronsky A., Kesari S., Lybeck M., Reid T.R. (2021). A review of persistent post-COVID syndrome (PPCS). Clin. Rev. Allergy Immunol..

[29] Plataki M., Hubmayr R.D. (2010). The physical basis of ventilator-induced lung injury. Expert Rev. Respir. Med..

[30] Cabrera-Benitez N.E., Laffey J.G., Parotto M., Spieth P.M., Villar J., Zhang H., Slutsky A.S. (2014). Mechanical ventilation–associated lung fibrosis in acute respiratory distress syndrome. Anesthesiology.

[31] Abodonya A.M., Abdelbasset W.K., Awad E.A., Elalfy I.E., Salem H.A., Elsayed S.H. (2021). Inspiratory muscle training for recovered COVID-19 patients after weaning from mechanical ventilation: A pilot control clinical study. Medicine.

[32] Umemura Y., Mitsuyama Y., Minami K., Nishida T., Watanabe A., Okada N., Yamakawa K., Nochioka K., Fujimi S. (2021). Efficacy and safety of Nintedanib for pulmonary fibrosis in severe pneumonia induced by COVID-19: An interventional study. Int. J. Infect. Dis..

[33] Wang F., Kream R.M., Stefano G.B. (2020). Long-term respiratory and neurological sequelae of COVID-19. Med. Sci. Monit..

[34] Esendağli D., Yilmaz A., Akçay Ş., Özlü T. (2021). Post-COVID syndrome: Pulmonary complications. Turk. J. Med. Sci..

[35] Ong K.-C., Ng A.W.-K., Lee L.S.-U., Kaw G., Kwek S.-K., Leow M.K.-S., Earnest A. (2005). 1-Year Pulmonary Function and Health Status in Survivors of Severe Acute Respiratory Syndrome. Chest.

[36] Torres-Castro R., Vasconcello-Castillo L., Alsina-Restoy X., Solis-Navarro L., Burgos F., Puppo H., Vilaró J. (2021). Respiratory Function in Patients Post-Infection by COVID-19: A Systematic Review and Meta-Analysis. Pulmonology.

[37] Gerardo A.M., Almeida T., Maduro S., Carvalho M., Boléo-Tomé J.P., Liberato H. (2021). Pulmonary function, functional capacity and health status in COVID-19 survivors. Rev. Med. Clínica.

[38] Afrin L.B., Weinstock L.B., Molderings G.J. (2020). COVID-19 hyperinflammation and post-COVID-19 illness may be rooted in mast cell activation syndrome. Int. J. Infect. Dis..

[39] Kritas S.K. (2020). Mast Cells Contribute to Coronavirus-Induced Inflammation: New Anti-Inflammatory Strategy. J. Biol. Regul. Homeost. Agents.

[40] Hafezi B., Chan L., Knapp J.P., Karimi N., Alizadeh K., Mehrani Y., Bridle B.W., Karimi K. (2021). Cytokine storm syndrome in SARS-CoV-2 infections: A functional role of mast cells. Cells.

[41] Kazama I. (2020). Stabilizing mast cells by commonly used drugs: A novel therapeutic target to relieve post-COVID syndrome?. Drug Discov. Ther..

[42] Kilinç E., Baranoğlu Y. (2020). Mast Cell Stabilizers as a Supportive Therapy Can Contribute to Alleviate Fatal Inflammatory Responses and Severity of Pulmonary Complications in COVID-19 Infection. Anadolu Klin. Tıp Bilimleri Derg..

[43] Kakavas S., Karayiannis D., Mastora Z. (2021). The Complex Interplay between Immunonutrition, Mast Cells, and Histamine Signaling in COVID-19. Nutrients.

[44] Baba A., Tachi M., Maruyama Y., Kazama I. (2015). Olopatadine Inhibits Exocytosis in Rat Peritoneal Mast Cells by Counteracting Membrane Surface Deformation. Cell. Physiol. Biochem..

[45] Kazama I., Saito K., Baba A., Mori T., Abe N., Endo Y., Toyama H., Ejima Y., Matsubara M., Yamauchi M. (2016). Clarithromycin Dose-Dependently Stabilizes Rat Peritoneal Mast Cells. Chemotherapy.

[46] Manniello M.D., Del Gaudio P., Aquino R.P., Russo P. (2017). Clarithromycin and N-acetylcysteine co-spray-dried powders for pulmonary drug delivery: A focus on drug solubility. Int. J. Pharm..

[47] Manniello M.D., Del Gaudio P., Porta A., Aquino R.P., Russo P. (2016). Aerodynamic properties, solubility and in vitro antibacterial efficacy of dry powders prepared by spray drying: Clarithromycin versus its hydrochloride salt. Eur. J. Pharm. Biopharm..

[48] (2021). The RECOVERY Collaborative Group Dexamethasone in Hospitalized Patients with COVID-19. N. Engl. J. Med..

[49] Cano E.J., Fonseca Fuentes X., Corsini Campioli C., O’Horo J.C., Abu Saleh O., Odeyemi Y., Yadav H., Temesgen Z. (2021). Impact of Corticosteroids in Coronavirus Disease 2019 Outcomes. Chest.

[50] Griesel M., Wagner C., Mikolajewska A., Stegemann M., Fichtner F., Metzendorf M.-I., Nair A.A., Daniel J., Fischer A.-L., Skoetz N. (2022). Inhaled Corticosteroids for the Treatment of COVID-19. Cochrane Database Syst. Rev..

[51] Milne S., Li X., Yang C.X., Leitao Filho F.S., Hernández Cordero A.I., Yang C.W.T., Shaipanich T., van Eeden S.F., Leung J.M., Lam S. (2021). Inhaled Corticosteroids Downregulate SARS-CoV-2-Related Genes in COPD: Results from a Randomised Controlled Trial. Eur. Respir. J..

[52] Maruta H., He H. (2020). PAK1-Blockers: Potential Therapeutics against COVID-19. Med. Drug Discov..

[53] Clemency B.M., Varughese R., Gonzalez-Rojas Y., Morse C.G., Phipatanakul W., Koster D.J., Blaiss M.S. (2022). Efficacy of Inhaled Ciclesonide for Outpatient Treatment of Adolescents and Adults with Symptomatic COVID-19: A Randomized Clinical Trial. JAMA Intern. Med..

[54] Ripa M., Galli L., Poli A., Oltolini C., Spagnuolo V., Mastrangelo A., Muccini C., Monti G., De Luca G., Landoni G. (2021). Secondary infections in patients hospitalized with COVID-19: Incidence and predictive factors. Clin. Microbiol. Infect..

[55] Langford B.J., So M., Raybardhan S., Leung V., Westwood D., MacFadden D.R., Soucy J.-P.R., Daneman N. (2020). Bacterial co-infection and secondary infection in patients with COVID-19: A living rapid review and meta-analysis. Clin. Microbiol. Infect..

[56] Hama Amin B.J., Kakamad F.H., Ahmed G.S., Ahmed S.F., Abdulla B.A., mohammed S.H., Mikael T.M., Salih R.Q., Ali R.K., Salh A.M. (2022). Post COVID-19 Pulmonary Fibrosis; a Meta-Analysis Study. Ann. Med. Surg..

[57] Echeverría-Esnal D., Martin-Ontiyuelo C., Navarrete-Rouco M.E., De-Antonio Cuscó M., Ferrández O., Horcajada J.P., Grau S. (2021). Azithromycin in the Treatment of COVID-19: A Review. Expert Rev. Anti Infect. Ther..

[58] Raghu G., Richeldi L. (2017). Current Approaches to the Management of Idiopathic Pulmonary Fibrosis. Respir. Med..

[59] Margaritopoulos G.A., Vasarmidi E., Antoniou K.M. (2016). Pirfenidone in the treatment of idiopathic pulmonary fibrosis: An evidence-based review of its place in therapy. Core Evid..

[60] Jin J., Togo S., Kadoya K., Tulafu M., Namba Y., Iwai M., Watanabe J., Nagahama K., Okabe T., Hidayat M. (2019). Pirfenidone Attenuates Lung Fibrotic Fibroblast Responses to Transforming Growth Factor-Β1. Respir. Res..

[61] Zhang F., Wei Y., He L., Zhang H., Hu Q., Yue H., He J., Dai H. (2022). A Trial of Pirfenidone in Hospitalized Adult Patients with Severe Coronavirus Disease 2019. Chin. Med. J..

[62] Liu Z., Ying Y. (2020). The inhibitory effect of curcumin on virus-induced cytokine storm and its potential use in the associated severe pneumonia. Front. Cell Dev. Biol..

[63] (2021). Memorial Sloan Kettering Cancer Center Phase II Study of N-Acetylcysteine in Severe or Critically Ill Patients with Refractory COVID-19 Infection. clinicaltrials.gov.

[64] Horowitz R.I., Freeman P.R., Bruzzese J. (2020). Efficacy of Glutathione Therapy in Relieving Dyspnea Associated with COVID-19 Pneumonia: A Report of 2 Cases. Respir. Med. Case Rep..

[65] Vitiello A., Troiano V., La Porta R. (2021). What will be the role of molnupiravir in the treatment of COVID-19 infection?. Drugs Perspect..

[66] Molnupiravir and Remdesivir Available for the Treatment of Non-Hospitalized COVID-19 Patients at High Risk of Progressing to Severe Disease. https://www.aifa.gov.it/en/-/disponibilit%C3%A0-molnupiravir-e-remdesivir-trattamento-pazienti-non-ospedalizzati-covid-19-1.

[67] Mitrani M.I., Bellio M.A., Meglin A., Khan A., Xu X., Haskell G., Arango A., Shapiro G.C. (2021). Treatment of a COVID-19 long hauler with an amniotic fluid-derived extracellular vesicle biologic. Respir. Med. Case Rep..

[68] Mitrani M.I., Bellio M.A., Sagel A., Saylor M., Kapp W., VanOsdol K., Haskell G., Stewart D., Abdullah Z., Santos I. (2021). Case report: Administration of amniotic fluid-derived nanoparticles in three severely Ill COVID-19 patients. Front. Med..

[69] Ampio Pharmaceuticals Receives Investigational Review Board Approval for Its Phase I Long COVID-19 Trial (AP-018). https://www.biospace.com/article/ampio-pharmaceuticals-receives-investigational-review-board-approval-for-its-phase-i-long-covid-19-trial-ap-018-/.

[70] Ragab D., Salah Eldin H., Taeimah M., Khattab R., Salem R. (2020). The COVID-19 cytokine storm; What we know so far. Front. Immunol..

[71] Abdellatif A.A.H., Tawfeek H.M., Abdelfattah A., El-Saber Batiha G., Hetta H.F. (2021). Recent updates in COVID-19 with emphasis on inhalation therapeutics: Nanostructured and targeting systems. J. Drug Deliv. Sci. Technol..

[72] Lechowicz K., Drożdżal S., Machaj F., Rosik J., Szostak B., Zegan-Barańska M., Biernawska J., Dabrowski W., Rotter I., Kotfis K. (2020). COVID-19: The potential treatment of pulmonary fibrosis associated with SARS-CoV-2 infection. JCM.

[73] Newman S.P. (2017). Drug delivery to the lungs: Challenges and opportunities. Ther. Deliv..

[74] Wright J. (2002). Inhaler Devices for the Treatment of Asthma and Chronic Obstructive Airways Disease (COPD). Qual. Saf. Health Care.

[75] Thorley A.J., Tetley T.D. (2013). New Perspectives in Nanomedicine. Pharmacol. Ther..

[76] Mahmud A., Discher D.E. (2011). Lung Vascular Targeting through Inhalation Delivery: Insight from Filamentous Viruses and Other Shapes. IUBMB Life.

[77] Sung J.C., Pulliam B.L., Edwards D.A. (2007). Nanoparticles for Drug Delivery to the Lungs. Trends Biotechnol..

[78] Brain J.D., Knudson D.E., Sorokin S.P., Davis M.A. (1976). Pulmonary Distribution of Particles given by Intratracheal Instillation or by Aerosol Inhalation. Environ. Res..

[79] Groneberg D.A., Witt C., Wagner U., Chung K.F., Fischer A. (2003). Fundamentals of Pulmonary Drug Delivery. Respir. Med..

[80] Borghardt J.M., Kloft C., Sharma A. (2018). Inhaled Therapy in Respiratory Disease: The Complex Interplay of Pulmonary Kinetic Processes. Can. Respir. J..

[81] Colombo P., Alhaique F., Caramella C., Conti B., Gazzaniga A., Vidale E. (2015). Principi di Tecnologia Farmaceutica.

[82] Labiris N.R., Dolovich M.B. (2003). Pulmonary Drug Delivery. Part I: Physiological Factors Affecting Therapeutic Effectiveness of Aerosolized Medications: Physiological Factors Affecting the Effectiveness of Inhaled Drugs. Br. J. Clin. Pharmacol..

[83] Newman S. (2014). Improving Inhaler Technique, Adherence to Therapy and the Precision of Dosing: Major Challenges for Pulmonary Drug Delivery. Expert Opin. Drug Deliv..

[84] O’Callaghan C., Barry P.W. (1997). The Science of Nebulised Drug Delivery. Thorax.

[85] Sou T., Bergström C.A.S. (2021). Contemporary Formulation Development for Inhaled Pharmaceuticals. J. Pharm. Sci..

[86] Pilcer G., Amighi K. (2010). Formulation Strategy and Use of Excipients in Pulmonary Drug Delivery. Int. J. Pharm..

[87] Rubin B.K., Fink J.B. (2005). Optimizing Aerosol Delivery by Pressurized Metered-Dose Inhalers. Respir. Care.

[88] Fink J.B. (2000). Metered-dose inhalers, dry powder inhalers, and transitions. Respir. Care.

[89] Ahookhosh K., Saidi M., Mohammadpourfard M., Aminfar H., Hamishehkar H., Farnoud A., Schmid O. (2021). Flow Structure and Particle Deposition Analyses for Optimization of a Pressurized Metered Dose Inhaler (PMDI) in a Model of Tracheobronchial Airway. Eur. J. Pharm. Sci..

[90] Chandel A., Goyal A.K., Ghosh G., Rath G. (2019). Recent Advances in Aerosolised Drug Delivery. Biomed. Pharmacother..

[91] Yawn B., Colice G. (2012). Practical Aspects of Inhaler Use in the Management of Chronic Obstructive Pulmonary Disease in the Primary Care Setting. COPD.

[92] Pothirat C., Chaiwong W., Phetsuk N., Pisalthanapuna S., Chetsadaphan N., Choomuang W. (2015). Evaluating Inhaler Use Technique in COPD Patients. COPD.

[93] van Beerendonk I., Mesters I., Mudde A.N., Tan T.D. (1998). Assessment of the Inhalation Technique in Outpatients with Asthma or Chronic Obstructive Pulmonary Disease Using a Metered-Dose Inhaler or Dry Powder Device. J. Asthma.

[94] Plaza V., Giner J., Rodrigo G.J., Dolovich M.B., Sanchis J. (2018). Errors in the Use of Inhalers by Health Care Professionals: A Systematic Review. J. Allergy Clin. Immunol. Pract..

[95] Lavorini F., Magnan A., Christophe Dubus J., Voshaar T., Corbetta L., Broeders M., Dekhuijzen R., Sanchis J., Viejo J.L., Barnes P. (2008). Effect of Incorrect Use of Dry Powder Inhalers on Management of Patients with Asthma and COPD. Respir. Med..

[96] Telko M.J., Hickey A.J. (2005). Dry Powder Inhaler Formulation. Respir. Care.

[97] Mehta P. (2016). Dry Powder Inhalers: A Focus on Advancements in Novel Drug Delivery Systems. J. Drug Deliv..

[98] Islam N., Gladki E. (2008). Dry Powder Inhalers (DPIs)—A Review of Device Reliability and Innovation. Int. J. Pharm..

[99] Roy A., Battle K., Lurslurchachai L., Halm E.A., Wisnivesky J.P. (2011). Inhaler Device, Administration Technique, and Adherence to Inhaled Corticosteroids in Patients with Asthma. Prim. Care Respir. J..

[100] Sumby B., Slater A., Atkins P.J., Prime D. (1997). Review of dry powder inhalers. Adv. Drug Deliv. Rev..

[101] Haughney J., Price D., Barnes N.C., Virchow J.C., Roche N., Chrystyn H. (2010). Choosing Inhaler Devices for People with Asthma: Current Knowledge and Outstanding Research Needs. Respir. Med..

[102] Heinen N., Klöhn M., Steinmann E., Pfaender S. (2021). In vitro lung models and their application to study SARS-CoV-2 pathogenesis and disease. Viruses.

[103] Seyfoori A., Amereh M., Dabiri S.M.H., Askari E., Walsh T., Akbari M. (2021). The role of biomaterials and three dimensional (3D) in Vitro tissue models in fighting against COVID-19. Biomater. Sci..

[104] Jenkins R.G., Moore B.B., Chambers R.C., Eickelberg O., Königshoff M., Kolb M., Laurent G.J., Nanthakumar C.B., Olman M.A., Pardo A. (2017). An official american thoracic society workshop report: Use of animal models for the preclinical assessment of potential therapies for pulmonary fibrosis. Am. J. Respir. Cell Mol. Biol..

[105] Kiener M., Roldan N., Machahua C., Sengupta A., Geiser T., Guenat O.T., Funke-Chambour M., Hobi N., Kruithof-de Julio M. (2021). Human-based advanced in vitro approaches to investigate lung fibrosis and pulmonary effects of COVID-19. Front. Med..

[106] Gerckens M., Alsafadi H.N., Wagner D.E., Lindner M., Burgstaller G., Königshoff M. (2019). Generation of human 3D lung tissue cultures (3D-LTCs) for disease modeling. JoVE.

[107] Sundarakrishnan A., Chen Y., Black L.D., Aldridge B.B., Kaplan D.L. (2018). Engineered cell and tissue models of pulmonary fibrosis. Adv. Drug Deliv. Rev..

[108] Liu G., Betts C., Cunoosamy D.M., Åberg P.M., Hornberg J.J., Sivars K.B., Cohen T.S. (2019). Use of precision cut lung slices as a translational model for the study of lung biology. Respir. Res..

[109] Hui K.P.Y., Cheung M.-C., Perera R.A.P.M., Ng K.-C., Bui C.H.T., Ho J.C.W., Ng M.M.T., Kuok D.I.T., Shih K.C., Tsao S.-W. (2020). Tropism, Replication Competence, and Innate Immune Responses of the Coronavirus SARS-CoV-2 in Human Respiratory Tract and Conjunctiva: An Analysis in Ex-Vivo and In-Vitro Cultures. Lancet Respir. Med..

[110] Martin C. (2021). Human lung slices: New uses for an old model. Am. J. Respir. Cell Mol. Biol..

[111] Bai Y., Krishnamoorthy N., Patel K.R., Rosas I., Sanderson M.J., Ai X. (2016). Cryopreserved human precision-cut lung slices as a bioassay for live tissue banking. A viability study of bronchodilation with bitter-taste receptor agonists. Am. J. Respir. Cell Mol. Biol..

[112] Sun A.M., Hoffman T., Luu B.Q., Ashammakhi N., Li S. (2021). Application of lung microphysiological systems to COVID-19 modeling and drug discovery: A review. Bio-Des. Manuf..

[113] Strikoudis A., Cieślak A., Loffredo L., Chen Y.-W., Patel N., Saqi A., Lederer D.J., Snoeck H.-W. (2019). Modeling of fibrotic lung disease using 3D organoids derived from human pluripotent stem cells. Cell Rep..

[114] Low L.A., Mummery C., Berridge B.R., Austin C.P., Tagle D.A. (2021). Organs-on-chips: Into the next decade. Nat. Rev. Drug Discov..

[115] Esch E.W., Bahinski A., Huh D. (2015). Organs-on-chips at the frontiers of drug discovery. Nat. Rev. Drug Discov..

[116] Si L., Bai H., Oh C.Y., Jin L., Prantil-Baun R., Ingber D.E. (2021). Clinically Relevant Influenza Virus Evolution Reconstituted in a Human Lung Airway-on-a-Chip. Microbiol. Spectr..

[117] Zhang M., Wang P., Luo R., Wang Y., Li Z., Guo Y., Yao Y., Li M., Tao T., Chen W. (2021). Biomimetic Human Disease Model of SARS-CoV-2-Induced Lung Injury and Immune Responses on Organ Chip System. Adv. Sci..

[118] Thacker V.V., Sharma K., Dhar N., Mancini G., Sordet-Dessimoz J., McKinney J.D. (2021). Rapid Endotheliitis and Vascular Damage Characterize SARS-CoV-2 Infection in a Human Lung-on-chip Model. EMBO Rep..

[119] Wu Q., Liu J., Wang X., Feng L., Wu J., Zhu X., Wen W., Gong X. (2020). Organ-on-a-chip: Recent breakthroughs and future prospects. BioMed. Eng. OnLine.

